# A novel task-based method for tracking social cognition and its association with social functioning in schizophrenia spectrum disorders: Preliminary findings

**DOI:** 10.3934/Neuroscience.2026010

**Published:** 2026-04-23

**Authors:** Anam A. Khan, Karmiella S. Ferster, Riaz B. Shaik, Tarik S. Bel-Bahar, Lauren Lepow, Jasmine Modasi, Faith Adams, Siddhartha Peri, Srinivasan Anantha Ramakrishnan, Matthew Cotter, Sarah E. MacPherson, R. Asaad Baksh, Cheryl Corcoran, Eva Velthorst, Muhammad A. Parvaz

**Affiliations:** 1 MAP Lab, Department of Psychiatry, Icahn School of Medicine at Mount Sinai, New York, NY, USA; 2 Human Cognitive Neuroscience, Department of Psychology, School of Philosophy, Psychology, and Language Sciences, University of Edinburgh, Edinburgh, UK; 3 Institute of Psychiatry, Psychology, and Neuroscience, Department of Forensic and Neurodevelopmental Sciences, King's College London, London, UK; 4 Department of Research, GGZ Noord-Holland-Noord, Heerhugowaard, Netherlands; 5 Department of Neuroscience, Icahn School of Medicine at Mount Sinai, New York, NY, USA; 6 Department of Artificial Intelligence & Human Health, Icahn School of Medicine at Mount Sinai, New York, NY, USA

**Keywords:** ESCoT, Theory of Mind, social cognition, schizophrenia spectrum disorder, interpersonal understanding of social norms, intrapersonal understanding of social norms, affective ToM, cognitive ToM, social functioning

## Abstract

Schizophrenia spectrum disorders (SCZ) are a group of psychiatric disorders that can severely impact social and occupational functioning. Social cognition plays a key role in social functioning, with deficits in social cognition potentially revealing social deficits. However, existing tests of social cognition are lengthy to administer and only measure one or two aspects of social cognition. The Edinburgh Social Cognition Test (ESCoT) is a newly developed brief assessment that evaluates multiple domains of social cognition, including cognitive Theory of Mind (ToM), affective ToM, and interpersonal and intrapersonal understandings of social norms. However, the ESCoT has not been utilized before to examine social cognition in SCZ. This observational study analyzed 18 individuals with SCZ and 19 healthy controls (HC), all of whom completed the ESCoT and several social functioning scales, including the Lubben Social Network Scale (LSNS), the Pinkham Social Skill Rating (PSSR), the Role Function Scale (RFS), and the Social Disconnectedness Scale (SDS). Between-group comparisons revealed significantly reduced scores in the participants with SCZ on cognitive and affective ToM but comparable scores on interpersonal and intrapersonal understandings of social norms, compared to HC. Additionally, the participants with SCZ showed reduced scores on the LSNS, PSSR, RFS, and SDS. Furthermore, ESCoT-derived cognitive ToM scores were positively correlated with scores on the LSNS, PSSR, and SDS, whereas affective ToM scores were positively correlated with the LSNS and PSSR. Interpersonal understanding of social norms was positively correlated with the LSNS and RFS score. The current study showed deficits in cognitive and affective ToM alongside other aspects of social functioning in SCZ compared to HC participants. The ESCoT sub-scores were correlated with scores from validated questionnaires of social functioning, thus validating the utility of the ESCoT to study social cognition in SCZ. Further investigation is recommended to replicate these findings in larger and more heterogeneous samples of SCZ individuals for a better generalization of these findings.

## 1. Introduction

Schizophrenia spectrum disorders (SCZ) are among the most disabling and economically burdensome psychiatric conditions [1]. SCZ is typically characterized by positive and negative symptoms, and impaired social and occupational functioning [2]. Positive symptoms are those experienced during psychotic episodes such as hallucinations, delusions, and disorganized thoughts or speech [3]. Negative symptoms denote the absence or lessening of aspects of thoughts, behaviors, and emotions that the person had before the illness, with examples including blunted emotional responses and the loss of ability to feel pleasure [4]. An impairment in social and occupational functioning is an important diagnostic feature of SCZ [5] and a major contributor to this disability burden. However, this impairment is often insufficiently addressed by typical pharmacological and behavioral treatments of SCZ [6].

Previous studies have shown that impairments in social functioning in SCZ are associated with reduced cognition; however, the amount of variance in social functioning accounted for by cognition has been inconsistent, ranging from 20% to 40% [7]–[9]. More recently, social cognition has been identified as another factor that affects social functional outcomes [10]. Social cognition is a multifaceted process that involves how people process, remember, and use information in social contexts to explain and predict behaviors of others [11], thus enabling them to navigate day-to-day interactions, and therefore requiring tasks with high social relevance and ecological validity. Subdomains of social cognition include theory of mind (ToM), emotion processing, social perception, and an understanding of social norms [12]. ToM describes the capacity to attribute mental states to others, cognitive ToM specifically deduces the thoughts and intentions of others, and affective ToM is the ability to discern others' emotions and feelings [13]. ToM deficits in SCZ have been extensively studied [14]–[17], and deficits, especially in cognitive and affective ToM within SCZ, have also been documented [18]–[20].

Similarly, the ability of individuals with SCZ to comprehend social norms from both interpersonal (i.e., how another person should behave) and intrapersonal (i.e., how a person themselves should behave) standpoints is under-researched, showing some deficits in these areas as well [21],[22].

Both the positive and negative symptoms of individuals diagnosed with SCZ have contributed to shortcomings in social interactions [23]. Given how much a human-centered world focuses on social interactions, these deficits add hurdles to those with a psychosis-spectrum diagnosis. When it comes to ToM, which is the ability to recognize another person's thoughts, actions, and emotional state, lacking or having limited insight into the social interpretations of another person may be an indicator of the severity of one's symptoms [24]. Previous literature has examined the specific link between ToM and those with a psychotic-disorder diagnosis, thereby finding evidence of limited social cognition in individuals that display some positive or negative symptoms of psychosis [15]. Furthermore, interpersonal and intrapersonal relationships, namely one's relationship between others and one's relationship within oneself, respectively, also contribute to one's social cognition [25]. To the best of our knowledge, these two aspects have not been looked at closely in SCZ; however, a large cohort study that examined the self-assessment of real-life functioning in patients with SCZ found a low overestimation of interpersonal relationships compared to their caregivers rating them [26], thereby offering an interesting outlook, as most literature express a significant relationship between ToM and clinical insights in SCZ [27]. It is with this understanding that this study's focus is on a measure of ToM that could be a better indicator of social deficits in those with a psychosis-spectrum diagnosis.

Although a variety of measures have been developed to assess social cognition [28]–[34], many existing tools are limited to specific domains or rely on static, language-dependent formats, decontextualized stimuli, or simplified task structures that only capture a narrow slice of real-world social understanding. Such approaches often fail to reflect the complexity, ambiguity, and contextual embeddedness of everyday social interactions, where individuals must integrate dynamic visual cues, social context, intentions, emotions, and social norms in real time. As a result, the need remains for a measure that can efficiently assess multiple facets of social cognition in a dynamic, ecologically grounded, and culturally generalizable way that more closely captures real-world social understanding.

The Edinburgh Social Cognition Test (ESCoT) [35] was developed to address this gap by operationalizing social cognition within naturalistic, context-rich social scenarios. ESCoT provides a multidimensional assessment of social cognition within a single, brief task. It evaluates four subdomains—cognitive ToM, affective ToM, interpersonal understanding of social norms, and intrapersonal understanding of social norms—through 11 short, animated scenarios that depict everyday social interactions. These scenarios are designed to reflect real-world social complexity, thereby requiring participants to integrate contextual information, infer mental states, interpret emotional cues, and evaluate norm-based behaviors within dynamic social situations. This structure more closely approximates the cognitive and interpretive demands of everyday social functioning than traditional laboratory-based social cognition tasks.

A major strength of ESCoT is that it doesn't rely on static images, isolated facial expressions, or textual vignettes. Instead, it uses brief animated videos that integrate visual, emotional, and contextual cues within coherent social narratives. By embedding social-cognitive judgments within realistic social interactions, ESCoT captures the complexity and contextual dependency of everyday social reasoning in a way that traditional decontextualized tasks cannot. Additionally, it is time-efficient (approximately 30 minutes) and independent of general intellectual ability and executive functioning [35], making it well suited for clinical populations in which cognitive load and fatigue can confound results. Furthermore, ESCoT has shown robust psychometric properties and demonstrated validity across diverse groups, including healthy adults [26], individuals with autism spectrum disorder [27], individuals with acquired brain injuries [28], and individuals with behavioral-variant dementia [29].

Despite its promise, ESCoT has not yet been applied in schizophrenia research. Evaluating its utility in this context could provide a more comprehensive and ecologically relevant understanding of social-cognitive deficits in SCZ, especially in cognitive and affective ToM and social-norm comprehension, and potentially identify novel treatment or rehabilitation targets.

In this current proof-of-concept study, we hypothesized that the ESCoT task will show social cognition deficits in individuals with SCZ, especially in cognitive and affective ToM and in the interpersonal understanding of social norms, but not in the intrapersonal understanding of social norms, compared to healthy control (HC). Furthermore, we hypothesized that more pronounced deficits in cognitive and affective ToM will be associated with poorer social functioning.

## 2. Methods

### 2.1. Participants

**Figure 1.**
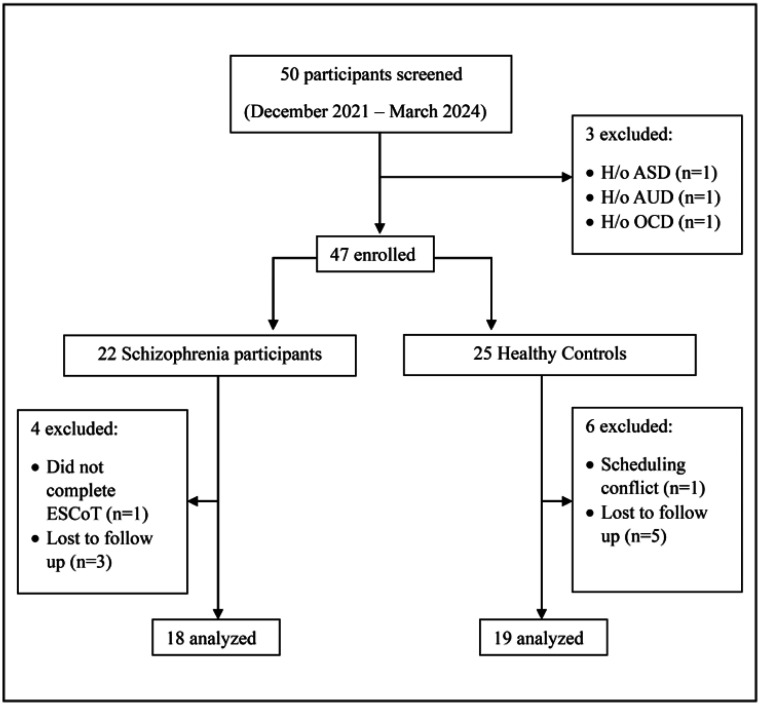
STROBE diagram. ASD: Autism Spectrum Disorder, AUD: Alcohol Use Disorder, ESCoT: Edinburgh Social Cognition Test and OCD: Obsessive Compulsive Disorder.

From December 2021 to March 2024, we recruited 50 individuals (Figure 1) who consented to participate in this study. The participants were recruited from large, ongoing research studies of SCZ. In these parent studies, the participants were recruited in a community-based outpatient setting, where psychiatric diagnoses were established using the DSM-5 criteria and confirmed via structured clinical interviews (Structured Clinical Interview for DSM Disorders; SCID). These assessments were conducted by trained research staff who completed formal in-person training by licensed psychologists and received direct supervision from their principal investigators.

For the SCZ participants, the inclusion criteria included the following: DSM-5 diagnosis of a SCZ based on structured clinical interview; age 18–50; sufficient English comprehension; and clinical/cognitive stability defined by the Brief Psychiatric Rating Scale (BPRS) conceptual disorganization ≤4 and MoCA > 15. The exclusion criteria included the following: clinically significant neurological disease (e.g., epilepsy); history of serious head injury (loss of consciousness >1 hour or neuropsychological sequelae); moderate or greater substance or alcohol use disorder within the past three months or diagnostic ambiguity due to substance use; intellectual or developmental disability (IQ < 70); current mood episode; and medical conditions that could interfere with social functioning (e.g., speech or hearing impairment, immobility).

For the HC participants, the inclusion and exclusion criteria were the same as those for the SCZ group, with the exception that the HC had no lifetime DSM-5 Axis I or II diagnosis.

To maintain clinical stability, all participants were required to have no changes in their medications 1 month prior to the study visit. Moreover, they were instructed to not take any sedatives or benzodiazepines within 12 hours of the assessments. All participants provided written informed consent to take part in this study, which was approved by the Institutional Review Board of the Icahn School of Medicine at Mount Sinai.

### 2.2. Measures

#### 2.2.1. Edinburgh Social Cognition Test (ESCoT)

The ESCoT [35] measures four aspects of social cognition within the same test: cognitive ToM, affective ToM, interpersonal understanding of social norms, and intrapersonal understanding of social norms. The ESCoT consists of 11 cartoon style videos (1 practice interaction, 5 interactions involved a social norm violation, and 5 portrayed everyday interactions that did not involve social norm violations) of everyday social interactions and takes about 30 minutes to administer. Each video was followed by 4 questions regarding the social interaction that assessed the different aspects of social cognition: one cognitive ToM question, one affective ToM question, one interpersonal ToM question, and one intrapersonal ToM question.

One of the videos showcased an interaction between a woman and a parking attendant, and the following questions were posed: “What is the woman thinking?”; “How does the woman feel at the end of the animation?”; “Did the parking attendant in the animation behave as other people should behave?”; and “Would you have acted the same as the parking attendant in the animation?”. Each response was scored from 0 to 3 based on the “quality” of the answer, with maximum points given for responses that, based on the question asked, extracted and integrated the relevant information from the video and included references to the social context in which the social interaction occurred. The authors of this instrument published guidelines for responses to this test to aid scorers. Per the template and using the interaction between a woman and a parking attendant, a score of zero for the cognitive theory of mind question – “What is the woman thinking?” – is one that does not acknowledge the parking attendant in the interaction, such as stating “She is thinking that she is going to get a ticket.” A 2-point score would mention the affective state in a cognitive question or does not give context despite mentioning the parking attendant as shown, such as “She is thinking that the parking attendant is going to give her ticket.” Alternatively, the 3-point score would mention both the parking attendant and the context of the interaction between the woman and the parking attendant, as demonstrated in the response “She is thinking that the parking attendant is going to give her ticket because she broke the rules.” Each question was scored by two trained lab members. Any score discrepancies were discussed, thereby utilizing the recorded video of the participant engaging in the ESCoT measure, and mutually resolved between the lab members. Every video could receive a maximum score of 12, resulting in a maximum total score of 120 points for the test. The scores for cognitive ToM, affective ToM, and interpersonal understanding of social norms were corrected for age.

#### 2.2.2. Scales to assess social functioning

To assess social functioning, we used the Lubben Social Network Scale – 6 (LSNS) [36], which is a self-report measure to assess social engagement with family and friends by asking about the quantity of their social networks and social support. Three questions regarding family relationships, such as “how many relatives do you feel close to such that you could call on them for help?”, and three questions regarding friendships, such as “how many of your friends do you see or hear from at least once a month?”, were asked. Each question was scored based on the following response: None = 0, One = 1, Two = 2, Three–Four = 3, Five through eight = 4, Nine or more = 5. The total score for the scale was obtained by adding all the individual scores and ranges between 0 and 30. Higher scores indicate more social engagement.

We used the Pinkham Social Skill Rating (PSSR) [37],[38] to assess interpersonal skills in individuals based on a video recording of an unstructured conversation between the research participant and a member of the research team that they have never met. The PSSR is based on a 9-point Likert scale, with higher scores representing better skills, thereby rating the overall interpersonal skills as well as various components of interpersonal skills such as clarity, fluency, meshing, appropriate affect, content, gaze, social anxiety, and engagement. The total score was obtained by adding all individual items, with 99 being the highest possible score.

Additionally, we used the Role Function Scale (RFS) [39] to measure the participant's level of functioning in daily life. It has four single rating scales, two of which specifically measure social functioning: Immediate Social Network (close friends/family) and Extended Social Network (neighborhood/community). Each scale is scored from 1 (represents a very minimal level of role functioning) to 7 (optimal level of role functioning) by asking about their relationships with members of their social network and the time spent with them. Then, both of the sub-scales scores were added to get the total RFS score that ranged from 2 to 14. Higher total scores denote higher social functioning, while lower scores denote lower functioning.

Lastly, we used the Social Disconnectedness Scale (SDS) [40] to understand social disconnectedness by measuring their network size, frequency of interaction and geographical proximity of network members, and the participation in social groups and volunteering. The total score reflects the severity of disconnectedness. Any score above zero signifies greater than average disconnectedness and scores below zero indicate less than average disconnectedness.

#### 2.2.3. Other scales/instruments

Additionally, we used the Brief Psychiatric Rating Scale (BPRS) [41] to track psychiatric symptoms, specifically those of Depression, Suicidality, Hostility, Guilt, Hallucination, Unusual Thought Content, and Conceptual Disorganization. Moreover, we used the Montreal Cognitive Assessment (MoCA) [42] to detect mild cognitive impairment. The Wide Range Achievement Test - Reading Test (WRAT-R) [43] subset was used to evaluate the recognition, naming, and decoding of isolated English letters and words. We selected the above battery of tests as each one of them have prior applications or psychometric support in samples with schizophrenia or serious mental illness, and together, they provide convergent and distinct indices of social functioning relevant to functional outcome in SCZ [5],[39],[44],[45].

### 2.3. Study design

This observational study was conducted at the Icahn School of Medicine at Mount Sinai and involved two sessions for a total of about 4 hours. The first session (~1 hr) was virtual to sign consent and to determine eligibility. The second session (~3 hrs) was an in-person visit to our research lab, where all the tasks were administered in a fixed order. The participants were compensated for their time and travel.

In the screening/first session, the participants engaged in a virtual Zoom call with two trained research team members. The participants reviewed and signed the consent form via REDCap. Their medical and psychiatric history was reviewed, BPRS was used to evaluate their psychosis and depression symptom severity, and the WRAT measured their basic reading skills. Then, eligible participants were scheduled for an in-person/second session.

During the in-person session, the participants completed a demographic questionnaire along with the PQB, LSNS, and SDS assessments. Unblinded research assistants, trained by licensed psychiatrist, administered the ESCoT, RFS and MoCA tests. Following these evaluations, a video-recorded conversation took place between the participant and a blinded member of the research team, who the participant had not interacted before. Then, the video of the interaction was used to score the PSSR by another blinded member who was not involved in participant recruitment or data acquisition.

### 2.4. Data analysis

All statistical analyses were performed using SAS 3.8.1 (Enterprise Edition) and SPSS (Version 29). Between-group differences in the demographic and clinical variables were assessed via a Chi-square test/Fischer's exact test for categorical variables and a Wilcoxon rank-sum test for continuous variables. Only variables which were significantly different between groups and significantly correlated with dependent variables (i.e., ESCoT scores) were considered as covariates for subsequent analyses. Additionally, to examine the association between the ESCoT scores and social functioning (e.g., LSNS, PSSR, RFS, and SDS), a Spearman's rank correlation was used. The alpha for all the statistical tests was set at p < 0.05. The False Discovery Rate (FDR) correction was used to adjust for multiple comparisons.

## 3. Results

### 3.1. Demographics

A total of 50 participants were screened for this study. Due to comorbid psychiatric conditions, 3 participants were excluded and 47 (22 SCZ and 25 HC) were enrolled. The final sample size used for the analysis was comprised of 18 individuals with SCZ and 19 HC, as more people were excluded due to reasons mentioned in Figure 1. The sample consisted of majority females (51%), non-Hispanics (75%), and Caucasians (33%). The groups significantly differed on the highest level of completed education (p = 0.005), employment status (p = 0.001), and income (p = 0.001). See Table 1 for details.

**Table 1.** Participant demographics and comparisons between healthy control and Schizophrenia group.

Demographic Characteristics	Healthy Controls (n = 19) Mean (SD) or %	Schizophrenia Group (n = 18) Mean (SD) or %	Test Statistic	p-value
Age in years	30.16(7.75)	32.56(6.94)	Z = 1.066	0.287
Biological Sex: Male/Female	42/58	56/44	χ² = 0.669	0.413
Ethnicity: Hispanic/non-Hispanic	21/79	29/71	P = 0.255	0.706
Race: White/Black/Other	47/32/21	18/35/47	P = 0.001	0.217
Highest level of completed education: 1 = some high school – 7 = Doctoral Degree	4.89(1.45)	3.35(1.54)	Z = −2.804*	0.005*
Mother's highest level of education: 1 = some high school – 7 = Doctoral Degree	4.47(2.01)	4.11(2.11)	Z = −0.562	0.574
Father's highest level of education: 1 = some high school – 7 = Doctoral Degree	4.74(1.88)	4.47(2.55)	Z = −0.546	0.585
Employment Status: Employed/Unemployed	74/26	18/82	χ² = 11.305*	0.001*
Income: 1 ≤ $2500 – 5 ≥ $100,000	2.21(1.23)	1.06(0.24)	Z = −3.342*	0.001*
Marital Status: Single/Married/Partner	68/11/21	100/0/0	P = 0.014*	0.034*

Note: P test statistic refers to Fischer's Exact Test, χ² test statistic refers to Chi square Test and Z test statistic refers to Wilcoxon Rank Sum Test. Continuous variable were reported in Means and Standard Deviations (SD) while Categorical variables were reported in percentages (%). * Statistically significant p value.

### 3.2. Clinical characteristics

Individuals in the SCZ group reported taking typical antipsychotics (22.22%), atypical antipsychotics (72.22%), mood stabilizers (50%), and antidepressants (27.78%), while only 4 participants from the HC group reported taking medications (Vitamins, Sildenafil, Allegra, and Isotretinoin). The SCZ group scored higher on the BPRS, with sub-scales measuring hallucinatory behavior (Z = 3.915, p < 0.001), unusual thought content (Z = 4.625, p < 0.005), depression (Z = 2.968, p = 0.005), and guilt (Z = 3.075, p = 0.004) showing significant group differences. No significant group differences were seen for the MoCA and WRAT scores.

### 3.3. Social cognition between groups

**Figure 2.**
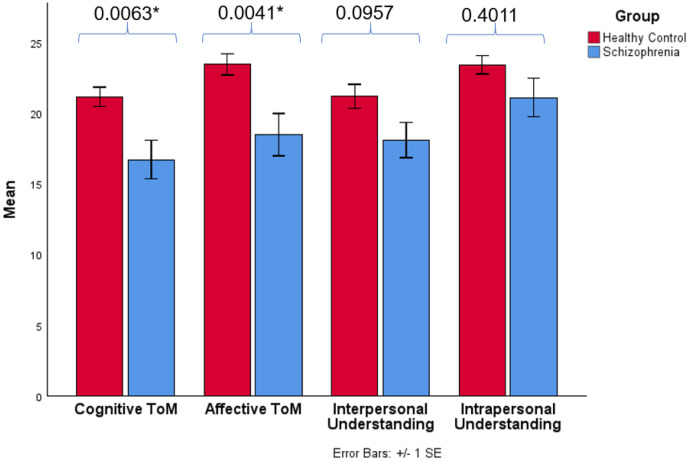
Bar graph showing mean ESCoT sub-scores by group along with p-value for each comparison. SCZ scored significantly lower on Cognitive ToM and Affective ToM, and marginally lower on interpersonal understanding of social norms compared to the HC, whereas no significant between-group difference was observed in scores of intrapersonal understanding of social norms. ESCoT: Edinburgh Social Cognition Test, HC: Healthy Controls, SCZ: Schizophrenia, and ToM: Theory of Mind.

Social cognition measured by the ESCoT sub-scores showed significant group differences based on the Wilcoxon rank-sum test for cognitive (Z = −2.73, p = 0.006) and affective (Z = −2.87, p = 0.004) ToM, with individuals with SCZ scoring significantly lower than HCs. Additionally, a trend for lower interpersonal understanding of social norms was observed in individuals with SCZ compared to the HCs (Z = −1.67, p = 0.096). The performance on the intrapersonal understanding of social norms in individuals with SCZ was at par with the performance in the HCs (Z = −0.84, p = 0.401) (Figure 2).

### 3.4. Social functioning between groups

**Figure 3.**
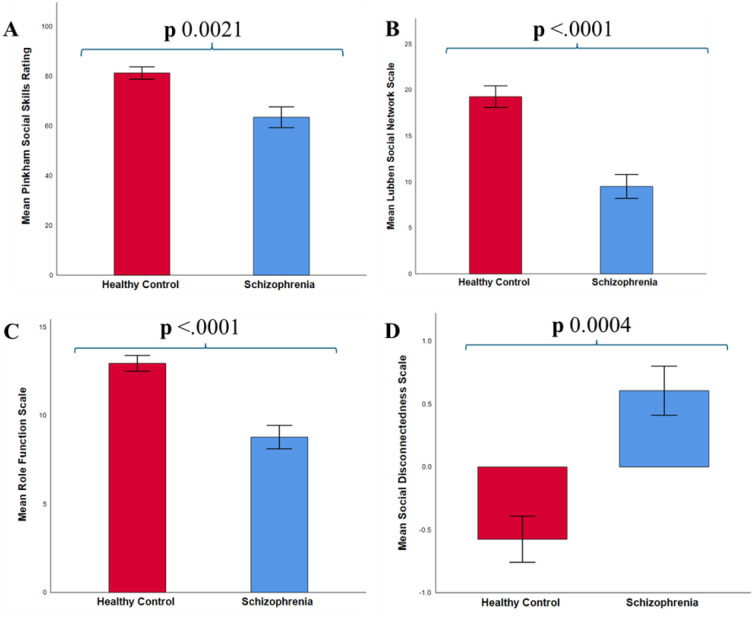
Bar graph showing group differences in PSSR (A), LSNS (B), SDS (C), and the RFS (D). Error bars: +/− 1SE

Expectedly, individuals with SCZ also showed significantly reduced social functioning compared to the HCs based on the scores of the LSNS (Z = −4.20, p < 0.001), PSSR (Z = −3.07, p = 0.002), RFS (Z = −4.02, p < 0.001), and SDS (Z = 3.56, p = 0.0004) scaled (Figure 3).

### 3.5. Association of social cognition with social functioning

**Figure 4.**
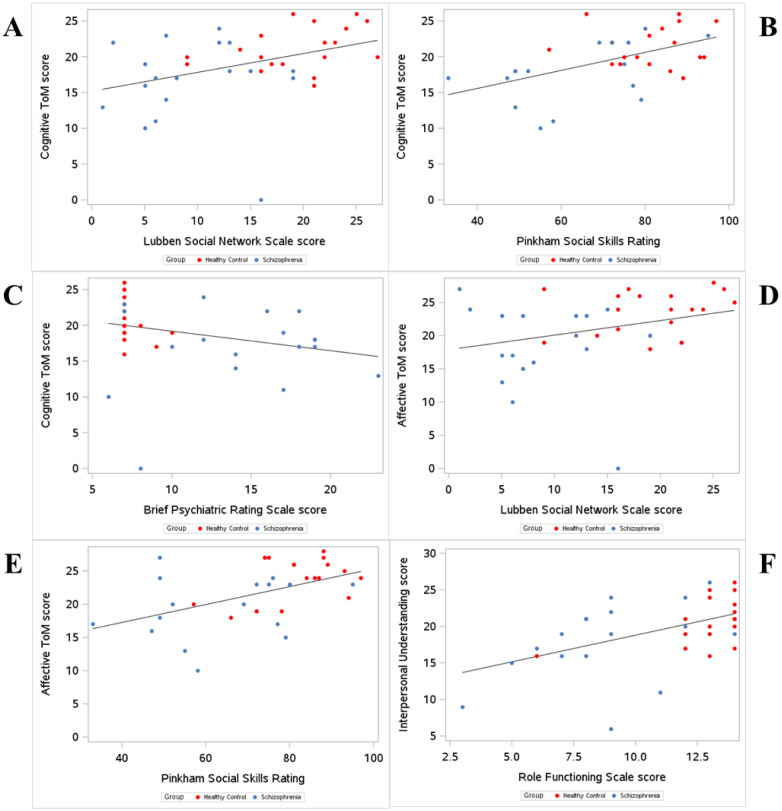
Spearman Correlation between ESCoT-derived scores on Cognitive ToM with LSNS (ρ = 0.441, p = 0.006) (A), PSSR (ρ = 0.496, p = 0.003) (B), and the BPRS (ρ = −0.433, p = 0.007) (C), between Affective ToM and LSNS (ρ = 0.364, p = 0.027) (D), PSSR (ρ = 0.493, p = 0.003) (E), and between interpersonal understanding score and RFS Score (ρ = 0.499, p = 0.002) (F). BPRS: Brief Psychiatric Rating Scale; ESCoT: Edinburgh Social Cognition Test; ToM: Theory of Mind.

The FDR-corrected Spearman rank correlations showed that the cognitive ToM sub-score of the ESCoT was positively correlated with the LSNS (ρ = 0.441, p = 0.006), PSSR (ρ = 0.496, p = 0.003) scores and was negatively correlated with the total BPRS score (ρ = −0.433, p = 0.007) in both groups. Additionally, the affective ToM sub-score was significantly positively correlated with the LSNS (ρ = 0.364, p = 0.027) and PSSR (ρ = 0.493, p = 0.003). Interpersonal understanding of social norms positively correlates with the RFS (ρ = 0.499, p = 0.002) (Figure 4). The performance on the ESCoT scores of intrapersonal understanding of social norms did not significantly correlate with any questionnaire measures of social functioning.

### 3.6. Association of social cognition with MoCA

The Spearman correlations revealed that the MoCA was moderately associated with cognitive ToM (ρ = 0.499, p = 0.0031), affective ToM (ρ = 0.360, p = 0.0392), and interpersonal norms (ρ = 0.550, p = 0.0012), but not with intrapersonal norms (ρ = 0.075, p = 0.7053). Moreover, the total ESCoT score showed a strong positive correlation with the MoCA (ρ = 0.609, p = 0.0003).

### 3.7. Association of social cognition with medication class

A sensitivity analysis was run using a Wilcoxon rank-sum test to examine whether the ESCoT performance differed as a function of medication class exposure in the SCZ group (n = 18). No significant differences in the ESCoT performance were observed between patients taking versus not taking each medication class, including key domains such as cognitive and affective ToM (e.g., typical antipsychotics: cognitive ToM Z = 0.11, p = 0.96; affective ToM Z = −0.74, p = 0.49; atypical antipsychotics: cognitive ToM Z = −1.33, p = 0.20; affective ToM Z = −0.35, p = 0.77; mood stabilizers: cognitive ToM Z = −0.22, p = 0.86; affective ToM Z = −1.28, p = 0.21; antidepressants: cognitive ToM Z = 1.63, p = 0.11; affective ToM Z = 1.38, p = 0.18).

### 3.8. Association of social cognition with socioeconomic status

We had significant group differences in socioeconomic status (such as education, occupation and income) (p < 0.005), which could influence performance on the ESCoT. Therefore, we ran a sensitivity analysis using education as a covariate in a univariate analysis of variance (ANOVA). We did not include all 3 variables (i.e., education, occupation and income) as covariates because they were highly intercorrelated (p < 0.001). After an adjustment for education, the difference between groups in cognitive ToM remained significant (p = 0.029), but reduced to a statistical trend in affective ToM (p = 0.068). Lastly, the group difference in the interpersonal norm did not survive the adjustment (p = 0.166) and the intrapersonal norm remained statistically non-significant (p = 0.321).

### 3.9. Post-Hoc power analysis

Post-hoc power analyses were conducted using G*Power 3.1 based on the observed effect sizes and sample sizes (SCZ n = 18; HC n = 19; α = 0.05). The between-group differences in the ESCoT subdomains demonstrated large effect sizes for cognitive and affective ToM (d = 0.97 and 0.99, respectively), which correspond to achieved power values of 0.82 and 0.83, respectively. The interpersonal and intrapersonal domains showed medium effect sizes (d = 0.68 and 0.51), with achieved power values of 0.52 and 0.33, respectively.

Post-hoc power analyses for correlations between the ESCoT sub-scores and social functioning measures (SCZ n = 18; HC n = 19; α = 0.05) revealed that moderate-to-large correlations, such as cognitive ToM with the LSNS (ρ = 0.441, power = 0.82), PSSR (ρ = 0.496, power = 0.92), and SDS (ρ = −0.433, power = 0.80), were well-powered, whereas smaller correlations, such as affective ToM with the LSNS (ρ = 0.364, power = 0.65), had limited power.

## 4. Discussion

The main objective of this proof-of-concept study was to examine the utility of the ESCoT in investigating social cognition in SCZ. The ESCoT is a brief test that assesses multiple domains of social cognition in a single test. Results from this study found that the participants with SCZ scored significantly lower on the cognitive and affective ToM, and marginally lower on the interpersonal understanding of social norms sub-score of the ESCoT compared to the HCs. Furthermore, whereas lower scores in both cognitive and affective ToM were associated with a lower social engagement with family and friends (i.e., lower LSNS score) and interpersonal skills (i.e., lower PSSR score), lower cognitive ToM was also differentially associated with clinical severity (i.e., higher BPRS score), thus highlighting the utility of ESCoT sub-scores in assessing both social functioning and symptom severity.

The preliminary findings of this study are the reduced scores in the participants with SCZ compared to the HC on cognitive and affective ToM sub-scores of the ESCoT. These results are consistent with previous literature that showed lower ToM in individuals with SCZ [14],[46],[47]. A meta-analysis [17] of results based on 29 studies comprised of 1500 participants found that individuals with SCZ performed at least one standard deviation below that of HC on ToM tasks. There has been limited research conducted that separately assesses cognitive and affective ToM in the same study, which further underscores the novelty of our results. The group difference in interpersonal understanding of social norms, though it did not reach statistical significance, showed marginally lower scores in the SCZ group compared to the HC. However, there was no significant difference in the intrapersonal understanding of social norms. These results are consistent with a previous study [48] that demonstrated that even though the SCZ group had a good understanding of socially appropriate behaviors and violations of social norms, their judgement of other people's social conduct was influenced by their ToM deficiencies. This could possibly explain the difference in the interpersonal and intrapersonal understanding of social norms in SCZ.

Expectedly, and consistent with prior literature, our results also showed that the group with SCZ had lower scores on validated scales of social functioning, such as the LSNS, PSSR, RFS, and SDS, compared to the HC. The associations between the ESCoT sub-scores and scores of these validated measures of social functioning highlights the utility of the ESCoT as a robust measure of social cognition in SCZ. Specifically, the cognitive and affective ToM scores were positively correlated with the LSNS and PSSR scores, thereby showing that poorer cognitive and affective ToM abilities are associated with worse social engagement with family and friends and interpersonal skills (i.e., poorer social functioning). Additionally, cognitive ToM scores negatively correlated with SDS scores, thereby showing that individuals with poorer cognitive ToM tend to be more socially isolated. The literature on the association between ToM and social functioning in SCZ is heterogenous. For example, some studies reported that ToM is not associated with social functioning in people with psychosis [49],[50], others have shown significant correlations between ToM and various measures of social functioning [51]–[53]. The use of different tools to assess ToM and social cognition may contribute to the mixed findings observed in previous research. Some of the social cognition tests used in these studies were the Hinting Task, the Picture Sequence Task, the Prosody Task, the Facial Expression of Emotions Task, and the Faux Pas Task. Many of these assessments require the participants to engage multiple aspects of their social cognition. However, distinguishing which specific aspect is being measured can be challenging. For instance, the Faux Pas task [54] necessitates the participants to draw upon their cognitive ToM, affective ToM, and understanding of social norms to recognize that the protagonist's feelings have been hurt due to a social norm violation, but it only gives a single score. In contrast, the Prosody task [55] solely relies on isolated speech samples without contextual information, thus failing to capture the complexities of real-world social interactions where individuals rely on nonverbal cues and contextual information to make judgments about others' mental states. Hence, the reasons for discrepancies in relationship between ToM and social functioning is the lack of ecological grounding and the challenge of distinguishing between the measured aspects of social cognition. This is where the ESCoT can be a valuable tool to assess social cognition in individuals with SCZ. The ESCoT employs videos that depict everyday social interactions, thereby providing context before and after each interaction to help the participants understand the content. It features distinct questions that assess four different facets of social cognition, thus enabling comparisons among these different facets. This makes the ESCoT a more comprehensive tool to evaluate social cognition in a manner that reflects real-world social interactions.

Scores on the interpersonal understanding of social norms were correlated with the LSNS and RFS scores, suggesting that a poorer understanding of social norms is associated with poorer social functioning, especially with social engagement with family and friends. These findings are consistent with findings from social skill training programs in SCZ [56] that target behaviors governed by social norms to improve the independent living skills in individuals with SCZ to better function in their communities.

Moreover, our results show that the ESCoT-derived scores, specifically of cognitive ToM, may be of clinical relevance, as individuals with greater BPRS scores showed lower cognitive ToM scores, even with the BPRS outliers excluded from the study. Such an association between ToM and clinical symptom severity has also been previously shown. Studies have shown that individuals with positive symptoms demonstrate over-mentalization (overly complex interpretation of the social clues), while individuals with disorganized symptoms showed reduced ToM (overly simplistic answer, despite intact capacity to represent mental states) [57]. Although, we only assessed positive symptoms in our study, future replication studies in larger samples could separately measure the association between type of symptom and deficits in cognitive and affective ToM.

Several additional findings warrant consideration. First, ESCoT sub-scores and total scores showed moderate positive correlations with the MoCA performance, thereby indicating partial overlap between general cognitive functioning and social-cognitive abilities. Importantly, no significant group differences were observed in the MoCA or WRAT scores, while robust ESCoT differences remained, thus supporting the specificity of social-cognitive impairment beyond global cognitive or literacy differences. Second, sensitivity analyses revealed no differences in the ESCoT performance as a function of medication class exposure, thus supporting the interpretation that the observed social-cognitive deficits reflect illness-related processes rather than pharmacological effects. Third, when education was included as a covariate, group differences in cognitive ToM remained statistically significant, while affective ToM was attenuated to a trend level and interpersonal norm differences were no longer significant. This pattern suggests that socioeconomic factors may partially influence the social-cognitive performance, while core cognitive ToM impairment remains robust. Post-hoc power analyses further contextualize these findings: while the study was adequately powered to detect moderate-to-large effects in cognitive and affective ToM domains and their associations with social functioning, interpersonal and intrapersonal norm domains demonstrated smaller effect sizes and lower achieved power, thus indicating that null and trend-level findings in these domains may reflect limited statistical power rather than true absence of effects.

Additionally, it is important to acknowledge the limitations of the current study. First, the sample size of this study was modest, thus limiting generalizability and the detection of smaller effects. Nonetheless, our sample was representative of typical SCZ samples, such that the age range for our participants were between 18 and 49 years, which covers the age for peak incidence of SCZ in men (20 to 24 years) and women (29 to 32 years) [58]. Additionally, the male to female ratio (1.3:1) was similar to that of previous studies [59], where males tend to have a slightly higher incidence ratio (1.4:1). Additionally, the SCZ group scored higher on the BPRS sub-scores and lower on the social functioning scales compared to HC. Second, all SCZ participants were referred from existing parent SCZ studies; therefore, the diagnoses were based on prior clinical assessments rather than full diagnostic assessment. Future work should include the Structured Clinical Interview for DSM-5 (SCID-5) [2] to confirm diagnosis and the Positive and Negative Syndrome Scale (PANSS) [60] to measure the symptom severity. The absence of comprehensive symptom severity measures restricts the interpretation of whether the ESCoT deficits are mediated by specific symptom dimensions. While the BPRS total and subscale scores were included and cognitive ToM showed associations with the overall symptom severity, these measures do not provide the dimensional specificity afforded by instruments such as the PANSS. Third, we did not collect data on the illness duration, which could potentially impact the social cognitive performance. Fourth, although the MoCA was used to assess the overall cognitive functioning, we recognize that it may not be sensitive to specific executive functioning and working memory deficits. Therefore, we are unable to attribute the deficits in the ESCoT outcomes to specifically reflect social-cognitive impairment or broader neurocognitive dysfunction. Therefore, future studies should include comprehensive neurocognitive batteries (e.g., MATRICS, BACS) to clarify the specificity of social-cognitive deficits and to examine the interplay between executive functioning and social cognition. Despite these limitations, our findings provide proof-of-concept evidence that supports the sensitivity of the ESCoT to social-cognitive deficits in schizophrenia.

Given the importance of social functioning impairments in diagnosing and predicting SCZ prognosis, it is crucial to measure factors that contribute to these impairments to develop appropriate treatments and monitoring progress. In this pilot study, we demonstrated the utility of using the ESCoT as a summary of various social cognition measures and a basis to examine social cognition in people with SCZ. There is evidence to suggest that the ESCoT is sensitive to social cognition impairments in SCZ and may be of research and clinical use. Future work should be done to replicate this in a larger and more diverse population.

## Use of AI tools declaration

The authors declare they have not used Artificial Intelligence (AI) tools in the creation of this article.
